# In vitro estrogenic, cytotoxic, and genotoxic profiles of the xenoestrogens 8-prenylnaringenine, genistein and tartrazine

**DOI:** 10.1007/s11356-021-12629-y

**Published:** 2021-02-01

**Authors:** Atefeh Nasri, Raimo Pohjanvirta

**Affiliations:** 1 Department of Food Hygiene and Environmental Health, University of Helsinki, Mustialankatu 1, FI-00790 Helsinki, Finland; 2 Department of Veterinary Biomedical Science, Western College of Veterinary Medicine, University of Saskatchewan, Saskatoon, Canada

**Keywords:** Phytoestrogens, Xenoestrogens, Estrogenic activity, Cytotoxicity, Genotoxicity, 8-prenylnaringenine, Genistein, Tartrazine

## Abstract

Phytoestrogens have been widely praised for their health-promoting effects, whereas synthetic environmental estrogens are considered a toxicological risk to human health. The aim of this study was therefore to compare in vitro the estrogenic, cytotoxic, and genotoxic profiles of three common estrogen-like endocrine-disrupting chemicals: the phytoestrogens 8-prenylnaringenine (8-PN) and genistein and the synthetic xenoestrogen tartrazine. As assessed by a yeast bioreporter assay and estrogen-dependent proliferative response in human mammary gland adenocarcinoma cell line (MCF-7), 8-PN showed the highest estrogen-like activity of the three compounds, followed by tartrazine and genistein. After 24-h incubation on MCF-7 cells, all three compounds exhibited low cytotoxicity in the lactate dehydrogenase assay and no genotoxicity in the micronucleus assay. These results demonstrate that 8-PN, genistein and tartrazine possess variable estrogenic activity but display little cellular toxicity in short-term tests in vitro. No difference between phytoestrogens and a synthetic xenoestrogen could be established.

**Supplementary Information:**

The online version contains supplementary material available at 10.1007/s11356-021-12629-y.

## Introduction

Chemicals known to interfere with the human endocrine system are classified as endocrine-disrupting chemicals (EDCs) (Roy et al. 1997). The structural similarity of certain categories of EDCs to estrogen allow them to act as estrogen mimics in the body, and they are therefore called estrogen-like endocrine-disrupting chemicals (EEDCs) (Roy et al. 2009). Major EEDCs, including natural and synthetic chemicals, are present in our environment and food. Among these EEDCs, some compounds are ingredients of plants and called phytoestrogens, while others are synthetic xenoestrogens (Lorand et al. 2010). In this study, we used 8-prenylnaringenine (8-PN) and genistein as examples of phytoestrogen and tartrazine as a representative of synthetic xenoestrogen.

Recent studies have revealed that beer can be a significant source of estrogenic activity to humans. This activity chiefly emanates from a prenylflavanone, 8-PN. 8-PN is also found as such in female hops (*Humulus lupulus L.*) that have been used for centuries as an essential raw material in beer brewing, providing bitterness and flavor to beer (Gerhauser et al. 2002; Rong et al. 2000). The estrogenic properties of hops have also been medically utilized in the treatment of gynecological disorders and in reducing hot flushes in menopausal women (Goetz 1990). Strikingly, the estrogenic activity of 8-PN in vitro has proven to be greater than that of established phytoestrogens such as coumestrol, genistein, and daidzein (Matsumura et al. 2005). In vitro and animal data have suggested that 8-PN has comparable binding activity to both estrogen receptor isoforms (ERα and ERβ). 8-PN can be produced from its precursor, xanthohumol, by intestinal microbial community of some, but not all, humans in large quantities, suggesting that even moderate beer consumption might be able to induce health effects due to increased serum levels of 8-PN (Possemiers et al. 2005).

Genistein is an isoflavonoid compound that is primarily found in soy products. This compound has structural similarity to 17β-estradiol and, therefore, can elicit estrogenic effects (Williamson-Hughes et al. 2006). Various in vitro and in vivo studies have demonstrated the estrogenic potency of dietary genistein. For example, genistein increased the expression of estrogen-regulated genes in estrogen-dependent cells such as human mammary gland adenocarcinoma cell line (MCF-7) in a dose-dependent manner (Allred et al. 2001). Soy products are often used as a replacement for hormone therapy with the perceived concept that the phytoestrogens they contain do not bear any risk for endocrine-related diseases, including breast cancer (Allred et al. 2001). However, it seems that genistein may exert proliferative or antiproliferative effects on cancer cells depending on the type of assays used, the timing of genistein administration, the level of endogenous estrogen, and the life stage and type of tumor (Patisaul and Jefferson 2010; Taylor et al. 2009). Although the findings on this topic have been incongruous, the previous notion of purely beneficial health impacts arising from genistein exposure has been challenged (EFSA 2009).

Tartrazine is an azobenzene and artificial yellow dye that is widely used in a variety of foodstuffs, drugs, and cosmetics. Together with related food dyes, it was suspected to cause hyperactivity and contribute to the attention deficit hyperactivity disorder (ADHD) in children, but the evidence is still inconclusive (Amchova et al. 2015). However, tartrazine has recently been reported to exert an estrogenic effect in vitro in the concentration range of 0.001-10 nM (Datta and Lundin-Schiller 2008; Axon et al. 2012), which is similar to the physiological concentration range of 17β-estradiol and within the human daily exposure range in, e.g., the USA today (Celojevic et al. 2011; FDA 2017). This is highly important because it suggests that tartrazine has the ability to interact with the estrogen signaling cascade at concentrations well below those which would be tested in classical toxicology studies. Therefore, further dose-response data of its toxicological and endocrinological effects are urgently needed.

Phytoestrogens have traditionally been praised due to their health-promoting effects on a wide variety of ailments including menopausal symptoms, skin aging, osteoporosis, cancer, and cardiovascular, neurodegenerative, immune, and metabolic diseases (Sirotkin and Harrath 2014). Synthetic xenoestrogens, in contrast, have been considered almost exclusively detrimental to human health due to their endocrine-disruptive capabilities (Paterni et al. 2017). However, evidence is emerging to challenge this dichotomous view. For example, a flavonoid was found to prolong estrus and suppress fertility after prenatal exposure in mice (Vaadala et al. 2019). Regarding genotoxicity and carcinogenicity, several convincing laboratory studies have reported the enhanced development of mammary tumors in animals treated with dietary phytoestrogens (Allred et al. 2001; Ju et al. 2006; Ju et al. 2002; Patisaul and Jefferson 2010). Bioflavonoids, including genistein, have also been shown to induce DNA double-strand breaks and promote genome rearrangements (Goodenow et al. 2020). These findings thus challenge the old concept of phytoestrogens as solely chemopreventive agents. However, this issue is still controversial. To clarify the matter, we set the hypothesis that the common phytoestrogens 8-PN and genistein cannot be distinguished from the synthetic xenoestrogen tartrazine based on their estrogenic as well as cyto- and genotoxic effects in vitro.

## Materials and methods

### Chemicals and labware

17β-estradiol, tartrazine, yeast nitrogen base without amino acids, D-luciferin, trisodium citrate dehydrate, yeast synthetic drop-out medium supplement, 3-(4,5-dimethylthiazol-2-yl)-2,5-diphenyltetrazolium bromide (MTT) reagent, L-leucine, L-tryptophan, L-histidine, adenine hemisulfate salt, sodium bicarbonate, fetal bovine serum (FBS), and charcoal-stripped FBS were purchased from Sigma-Aldrich (Saint Louis, USA). Progesterone, 8-PN, tamoxifen, and cytochalasin B were obtained from Cayman Chemical (Ann Arbor, MI USA), while genistein was obtained from LC Laboratories (Woburn, MA, USA). Dulbecco’s Modified Eagle Medium (DMEM) without or with phenol red, cell culture-grade L-glutamine, and trypsin-EDTA solution were obtained from Gibco®, ThermoFisher Scientific (Paisley, Scotland, UK). Giemsa dye was purchased from Merck (Darmstadt, Germany).

17β-estradiol, progesterone, tamoxifen, 8-PN and genistein were dissolved in ethyl alcohol (EtOH) or dimethyl sulfoxide (DMSO), and the stock solutions were kept in a refrigerator for further analysis. Tartrazine was dissolved in water. D-luciferin was dissolved directly in citrate buffer and stored in a freezer at −18°C. Cytochalasin B was dissolved in DMSO and kept in a freezer at −18°C. MTT reagent was dissolved in phosphate-buffered saline (PBS) and then filtered through 0.2-μm syringe filters. The filtered MTT dye was stored in opaque tubes in a freezer at −18°C. The final concentration of all the vehicles in cell culture-based assays was lower than 0.1%.

Cell culture flasks, cell culture dishes, transparent cell culture plates (12 and 96 wells), and white 96-well flat-bottom plates were purchased from CELLSTAR®, Greiner Bio-One GmbH (Kremsmünster, Austria).

### Yeast bioluminescent assay

Yeast bioluminescent assay was performed as previously described (Omoruyi and Pohjanvirta 2018). Two recombinant yeasts, *Saccharomyces cerevisiae* BMAEREluc/ERα and *Saccharomyces cerevisiae* BMA64/luc, were kindly provided by Dr. Johanna Rajasärkkä, Department of Food and Environmental Sciences, Faculty of Agriculture and Forestry, University of Helsinki. In the BMAEREluc/ERα, human ERα is expressed. After estrogen-like chemicals bind to ERα, the dimerized receptor binds to a specific DNA sequence called estrogen response element (ERE), in the promotor region of the *luc* reporter gene, inducing its expression. In BMA64/luc, the luciferase is expressed constitutively, and this strain can be used for the evaluation of cytotoxicity of test compounds.

Both strains were revived from glycerol stock solution on agar and broth forms of synthetic complete medium supplemented with the required amino acids. After the absence of contamination was confirmed by observing single colonies’ morphology on agar plate, approximately 10 μL of cultured yeasts in broth was transferred to 4 mL of synthetic defined (SD) medium supplemented with the required amino acids. The culture was incubated at 30°C on a shaker incubator (200 rpm). After 24-h incubation, the culture was diluted with SD broth to reach the optimal density (OD600) of 0.4 CFU/mL. Subsequently, the culture was incubated in the same incubation condition until the OD600 reached 0.6–0.7 CFU/mL.

Next, 90 μL of cultured yeasts containing 1:20 luciferin solution (0.042 g luciferin dissolved in 9.75 mL of 0.2 M trisodium citrate, and 5.525 mL of 0.2 M citrate, pH 5) was pipetted into every well of a white 96-well flat-bottom plate, and 10 μL of test compounds in a defined concentration range (diluted in 5% EtOH) were added. The plate was incubated at 30°C for approximately 2.5 h, and was then shaken briefly. The luminescence measurement was performed with Luminoskan Ascent microplate luminometer (Thermo Labsystems) using the recommended settings as follows: 1000 ms integration time, single measurement mode, shaking at 60 rpm for 5 s prior to measurement.

A total of 5% EtOH was used as the vehicle; progesterone, as the negative control; 17β-estradiol, as the positive control; and tamoxifen, as an inhibitor of estrogen-induced response in the yeast strain expressing ERα. Each concentration of test compounds and controls in the assay was tested in triplicate, and the experiment was performed twice.

The sigmoidal luminescence emission curves against increasing concentrations of 17β-estradiol, 8-PN, genistein, and tartrazine were obtained with the GraphPad Prism software (GraphPad Software, Inc., Prism 8 for Windows, Version 8.4.2, San Diego, CA USA). The fold induction corrected (FIC) and limit of detection (LOD) were calculated based on the methods described before (Leskinen et al. 2005). The EC_50_ values for estrogenic potency of 17β-estradiol, 8-PN, genistein, and tartrazine were calculated based on fitted dose-response curves using three-parameter nonlinear logistic regression with the coefficient of determination (*R*^2^) within the range of 0.88–0.91.

### Cell culture

MCF-7 cell line which is sensitive to estrogen (ERα^+^) was kindly provided by Dr. Sari Tojkander (Department of Veterinary Biosciences, Faculty of Veterinary Medicine, University of Helsinki). Cells were usually grown in T25 or T75 flasks containing DMEM with or without phenol red and supplemented with 7–10% FBS and 4 mM L-glutamine at 37°C in 5% CO_2_/95% humidified atmosphere. In order to eliminate the disturbing effect of hormones usually present in FBS, cell culture medium supplemented with commercially available charcoal-stripped FBS and 4 mM L-glutamine (SF medium) was used for experiments.

### Cell proliferation assay

The effect of EEDCs on the proliferation of MCF-7 cells was assessed as previously described by measuring the capacity of the cells in each well to reduce MTT reagent (Van Meeuwen et al. 2007). Approximately 5000 cells/200 μL of SF medium containing DMEM without phenol red, 5% charcoal-stripped FBS, and 4 mM L-glutamine were seeded per well in a 96-well cell culture plate. On day 2, cells were treated with the SF medium containing test compounds or vehicle. The final concentration of the solvents DMSO and EtOH was lower than 0.1%. On day 5, the SF medium containing test compounds or vehicle were refreshed. On day 8, cells were washed with PBS twice and then incubated with the SF medium containing 1 mg/mL MTT reagent for 1 h. The ability of MCF-7 cells to reduce MTT was measured at the reference wavelength of 595 nm using a microplate reader (Multiskan Ascent, Thermo Fisher Scientific). All the exposures were in triplicate.

In order to verify that the proliferative effect of test compounds was ER-mediated, tamoxifen, as an inhibitor of estrogen-induced response, was used. The dose-response curves of test samples were plotted against logarithmic-transformed concentrations. The EC_50_ value for EEDCs was calculated from the equation obtained by non-linear regression curve fitting, with *R*^2^ being 0.88–0.94.

### Cytotoxicity test

Cytotoxicity was assessed by lactate dehydrogenase (LDH) release. For LDH measurement, Pierce LDH Cytotoxicity Assay Kit (Thermo Scientific, Rockford, USA) was used. MCF-7 cells were seeded at 2.8×10^5^ in a transparent 96-well plate, and corner cells of the plate were filled with PBS to prevent the edge effect. SF medium containing DMEM without phenol red, 1% charcoal-stripped FBS, and 4 mM L-glutamine were used for this assay. Plates were incubated for 24 h, and then the cells were treated with test compounds at concentrations ranging from 10^−14^ to 10^−4^ M or vehicle for another 24 h. On day 3, culture supernatant was collected in a separate 96-well plate, and the assay was continued according to the manufacturer’s instructions. Final absorbance was measured with the microplate reader at 490 and 680 nm. The final absorbance was corrected with background absorbance at 680 nm, and cytotoxicity was calculated as suggested by the kit’s manufacturer. Vehicle control was 0.1% EtOH or DMSO. All the exposures were in triplicate, and the experiment was carried out twice.

### In vitro micronucleus assay for cytokinesis block

The genotoxicity of EEDCs to MCF-7 cells was assessed by the in vitro micronucleus assay for cytokinesis block. MCF-7 cells were seeded at a density of 5×10^4^/well in a 12-well plate in SF medium containing DMEM, 7% charcoal-stripped FBS and 4 mM L-glutamine. These cells were treated with test compounds or the positive control for 24 h. Then, cells were washed twice with PBS, treated with 2 μg/mL cytochalasin B, and incubated for 1.5–2 normal cell cycle lengths (~24 h for MCF-7 cells). Finally, cells were washed with PBS and fixed with 4% paraformaldehyde for 10–15 min (in PBS, pH 6.9). Before staining cells with 5% Giemsa stain for 20 min, plates were washed again with PBS and dried. The frequency of micronuclei per 1000 binucleated cells was counted for each well, and the ratio of micronucleus frequency in treated vs. control cells was defined as fold induction. Cells in mitosis were omitted.

### Data analysis

The statistical analysis was performed using the SPSS statistical software (IBM SPSS Statistics for Windows, Version 23.0., Armonk, NY, USA) or GraphPad Prism (GraphPad Software, Inc., Prism 8 for Windows, Version 8.4.2, San Diego, CA, USA). The normality of data distribution was analyzed by Shapiro-Wilk’s test, and all data proved normally distributed. The significance level was set at *p* < 0.05. Dose-response data from yeast bioluminescent and cell proliferation assays were analyzed with GraphPad Prism using three-parameter nonlinear logistic regression. Furthermore, data obtained from LDH cytotoxicity test and in vitro micronucleus assay were statistically assessed by the Cochran-Armitage test for trend in proportions.

## Results

### Yeast bioluminescent assay

**Fig. 1:**
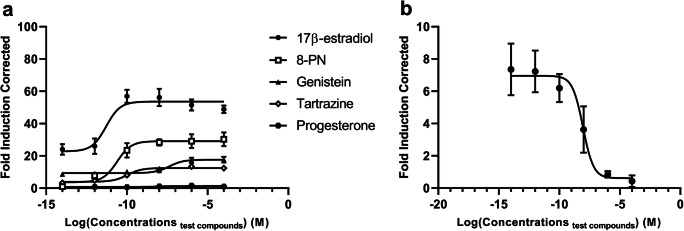
Dose-estrogenic response curve of EEDCs, 17β-estradiol, progesterone (**a**) and tamoxifen in the presence of 10^−12^ M of 17β-estradiol (**b**). All the concentrations are presented in a logarithmic (log_10_) form. Values represent the mean ± range of two separate experiments. Each experiment was performed in triplicate

The yeast bioluminescent assay was employed to investigate the estrogenic potency of the three test compounds. In *S. cerevisiae* BMAEREluc/ERα, all these EEDCs along with 17β-estradiol produced a sigmoidal dose-response curve, and the value of *R*^2^ was always close to 1 (Fig. 1a). 17β-estradiol was used as a positive control, and it had the lowest EC_50_ value of around 0.005 nM. The second highest estrogenic potency was exhibited by 8-PN with an EC_50_ value of 0.02 nM, followed by tartrazine and genistein (EC_50_ values of 0.1 nM and 40 nM, respectively).

As also evident from Fig. 1a, the negative control compound progesterone did not produce any detectable luciferase activity. In contrast to all other compounds tested, tamoxifen displayed an inverse dose-response: as its concentration increased, the FIC value decreased (Fig. 1b). Altogether, these data confirm that the response was mediated by ERα. The LOD in this assay was 3.77 FIC.

Another yeast strain, *S. cerevisiae* BMA64/luc (which displays constitutive luciferase expression), was used to monitor the cytotoxicity of the test compounds. In the original publication describing this method, the authors had used 10% DMSO as the solvent control (Leskinen et al. 2005). However, in our hands, even 5% DMSO proved to be a cytotoxic vehicle for the compounds (Supplementary Table S1). We therefore resorted to 5% ethanol, which did not display any cytotoxicity with the test compounds (Supplementary Table S2).

### Cell proliferation assay

**Fig. 2:**
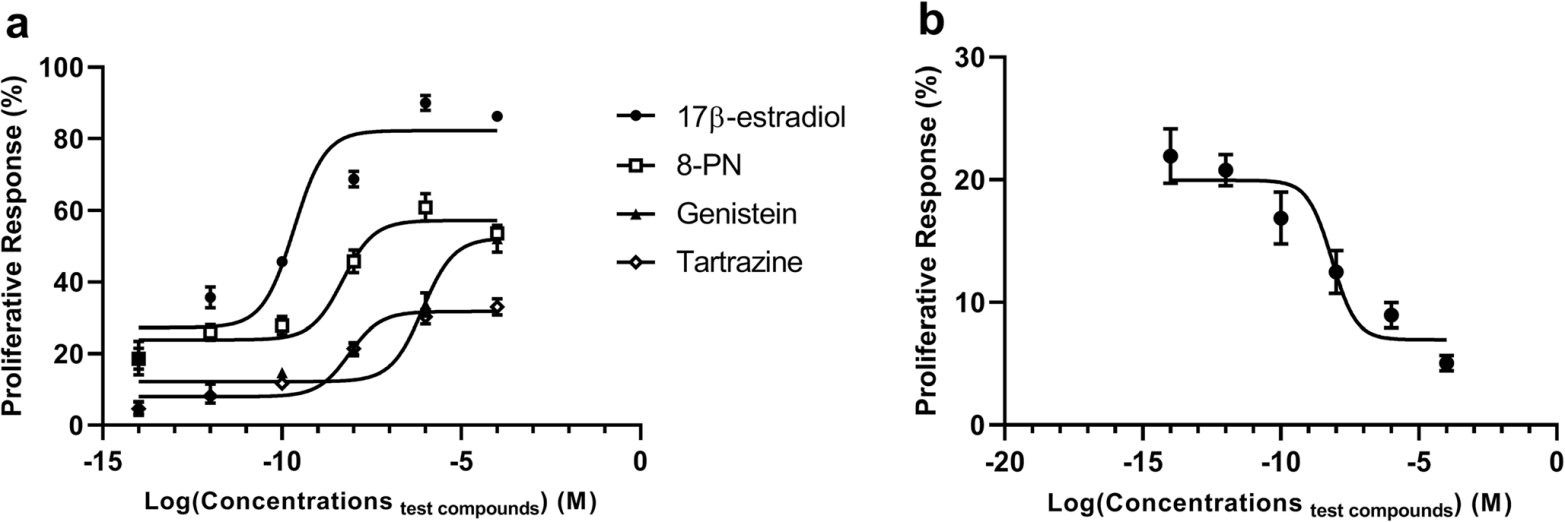
The proliferative effect of 17β-estradiol, three EEDCs (**a**) and tamoxifen in the presence of 10^−12^ M of 17β-estradiol (**b**) on MCF-7 cells after 24-h treatment measured as the ability of cells to reduce the MTT reagent. All the concentrations are presented in a logarithmic (log_10_) form. The symbols and error bars depict the mean and range of two separate experiments. Each experiment was performed in triplicate

We next evaluated the proliferative effect of the test compounds in estrogen-dependent MCF-7 cells grown on hormone-depleted medium in order to eliminate the interference of other hormones. Unsurprisingly, 17β-estradiol showed the highest efficacy (maximum induction of cell growth) and potency (EC_50_ of 0.21 nM) in this assay (Fig. 2a). The efficacies of 8-PN and genistein were about half and that of tartrazine about one third compared with 17β-estradiol. However, the rank order for potency after 17β-estradiol was 8-PN (EC_50_= 4.98 nM) > tartrazine (EC_50_= 7.46 nM)> genistein (EC_50_= 831 nM). Again, tamoxifen displayed an inverse dose-response curve (Fig. 2b), implying that also this response was mediated by ERα.

### LDH cytotoxicity test

**Fig. 3:**
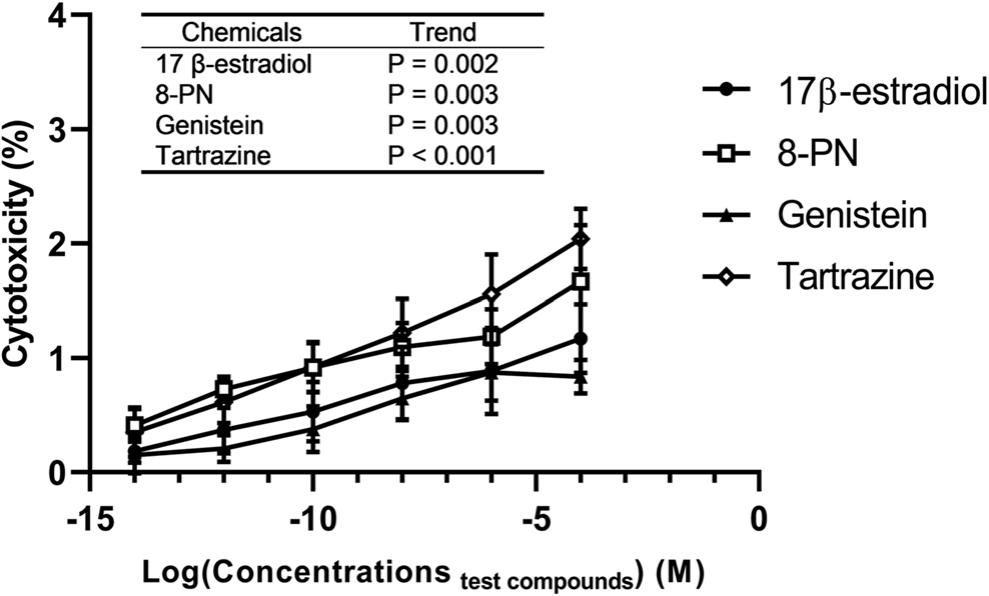
The cytotoxic effect of 17β-estradiol and three EEDCs on MCF-7 cells after 24-h exposure (percentage of all cells). The concentrations are provided in a logarithmic (log_10_) form. Values represent the mean and range of two separate experiments. Each experiment was performed in triplicate

The cytotoxic effect of 17β-estradiol, 8-PN, genistein, and tartrazine was assessed on MCF-7 cells (Fig. 3). All these compounds showed a statistically significant linear trend for increasing cytotoxicity as a function of concentration up to the highest concentration (10^−4^ M) tested (*p*<0.05). However, the maximum level of cytotoxicity was less than 2.5% indicating that these chemicals exert only a weak cytotoxic effect on MCF-7 cells.

### Micronucleus assay

The total number of micronuclei per 1000 binucleated MCF-7 cells exposed to 17β-estradiol, 8-PN, genistein, tartrazine, and benzo[a]pyrene for 24 h is shown in Table 1. The concentration of the positive control, benzo[a]pyrene, was 10–100-fold lower than that of the test compounds. A slight dose-dependent tendency to increase the occurrence of micronuclei in MCF-7 cells was observed for the three EEDCs (particularly tartrazine), but it did not quite reach statistical significance (*p* > 0.05).

**Table 1:** The effect of xenoestrogens and 17β-estradiol on micronucleus formation in MCF-7 cells

Group	Conc (M)	Micronuclei per 1000 binucleated cells*					Fold	Trend
		Replicates			Average	SD		
Benzo[a]pyrene	10^−9^	91	86	94	90.33	4.04	8.74	
Untreated		11	12	8	10.33	2.08		
Vehicle control	0.1% EtOH	7	10	13	10	3		
17 β-estradiol	10^−8^	12	14	13	13	1	1.26	*p* = 0.91
	10^−7^	10	13	17	13.33	3.51	1.29	
8-PN	10^−8^	16	19	21	18.67	2.52	1.81	*p* = 0.32
	10^−7^	24	20	23	22.33	2.08	2.16	
Genistein	10^−8^	14	16	16	15.33	1.15	1.48	*p* = 0.61
	10^−7^	17	15	19	17	2	1.65	
Tartrazine	10^−8^	21	19	25	21.67	3.06	2.10	*p* = 0.07
	10^−7^	29	31	27	29	2	2.81	

*Values represent the mean ± SD of three replicates in a single experiment

## Discussion

In this study, we have evaluated the estrogenic, cytotoxic, and genotoxic profiles of selected natural and synthetic EEDCs that are structurally similar to 17β-estradiol_._ We investigated the estrogenic capacity of 8-PN and genistein as representatives of phytoestrogens and tartrazine as a synthetic xenoestrogen in common use by a yeast bioluminescent assay and MCF-7 cell proliferation assay. Results from both assays indicated that these compounds can activate the ERα in vitro and consequently may cause some physiologic alterations similar to estrogen, such as proliferation in an estrogen-dependent cell line. These tests are believed to reflect the true estrogenicity of the compounds tested, because both yeast strains respond to natural and synthetic estrogens in the same manner (Schaefer et al. 2003), and in the cell proliferation assay, the total number of viable cells in a hormone-deprived medium is directly related to the estrogenic effect of test substances (Zierau et al. 2008). Although yeast-based assays have often been reported to have lower sensitivity than in vitro mammalian cell-based assays (Overk et al. 2008; Zierau et al. 2002), in our study, the yeast assay proved more sensitive than the MCF-7 cell proliferation assay based on the EC_50_ values recorded for the positive control and test compounds. This outcome is in agreement with a previous study which used a reporter gene construct in human ERα-positive MCF-7 cells and determined the EC_50_ concentration for estrogen receptor activation by tartrazine to be 160 nM (Axon et al. 2012), i.e., two orders of magnitude higher than in our yeast assay. Here, both the yeast reporter and MCF-7 cell proliferation assay indicated that 8-PN possesses the highest estrogenic potency of the three xenoestrogens tested, although it, too, lagged behind the natural estrogen 17β-estradiol in this regard. Similarly, tartrazine was ranked third in estrogenic potency by both assays, followed by genistein. This congruency lends support to the reliability of the assays and demonstrates that they are a valid tool for assessment of estrogenic activity of xenobiotics.

8-PN has been recognized as one of the most potent natural estrogen-like compounds in vitro and in vivo (Schaefer et al. 2003; Zierau et al. 2008). 8-PN demonstrated high relative binding affinity for ERα in cell-free competitive binding assays, in which purified human ERα and ERβ were used (Roelens et al. 2006). In our experimental assays, 8-PN also consistently exhibited the highest estrogenic capacity. The previously reported high potency of 8-PN in inducing estrogenic responses in MCF-7 and the human transgenic MVLN cell lines upon binding to the ERs is in keeping with our results (Helle et al. 2014; Zierau et al. 2002).

Genistein has also demonstrated estrogenic capacity in vitro and in vivo. The majority of initial studies (especially in vivo on mouse and rat uterus) revealed the estrogenic propensity of this compound (Cotroneo et al. 2001; Diel et al. 2001; Jefferson et al. 2007; Santell et al. 1997). Dietary genistein hindered the regression of mammary tissues, stimulated the secretion of prolactin, increased the wet and dry weight of uterus, and enhanced the uterine expression of the *c-fos* gene in ovariectomized rats (Santell et al. 1997). Subsequent in vitro studies using the luciferase reporter gene assay in MCF-7- and HeLa-derived cell lines (MVLN and HGELN, respectively), MCF-7 proliferation assay, and yeast bioluminescent assay confirmed the estrogenic potency of this compound (Gutendorf and Westendorf 2001). In agreement with our present findings, genistein was reported to have lower estrogenic potency than 8-PN (Takamura-Enya et al. 2003).

Tartrazine has been classified as a xenoestrogen that can bind to ERα in the MCF-7 cell line (Axon et al. 2012). It was found to induce a proliferative effect in ER^+^-T47D breast cancer cells and augment the expression of an estrogen reporter gene (Datta and Lundin-Schiller 2008). In our study, tartrazine similarly exhibited a proliferative effect on MCF-7 cells with EC_50_ of 7.46 nM. This raises the question whether tartrazine could create any estrogenic effect in humans at its prevailing serum concentrations. Based on the data published by EFSA, the 95th or 97.5th percentile exposure levels for human is 7.3 mg/kg body weight/day (EFSA 2009). If we assume that this dose is taken as bolus; only 2% can be absorbed and will be available in our body; the compound is evenly distributed throughout the body; and the maximum concentration is reduced by 50% due to absorption, metabolism, and elimination, we end up at a serum concentration of 137 nM. This level exceeds the EC_50_ value obtained in the present study, thus suggesting that dietary intake in highly exposed people may be sufficient to trigger some estrogenic effects by this compound.

There has been an information gap regarding the cytotoxic and genotoxic capacity of 8-PN. This compound and the majority of its derivates were reported to show weak cytotoxicity in human acute myeloid leukemia (HL-60) cells (Allsopp et al. 2013). Strikingly, 8-PN in a dose range (0.001–20 μM) and its precursor at a low dose (0.01 μM) improved mitochondrial function and decreased ROS production of MCF-7 cells (Blanquer-Rosselló et al. 2013). These results are in agreement with our observation of low cytotoxicity and the absence of genotoxicity for 8-PN.

Genistein was found to induce micronuclei in the mouse lymphoma L5178Y cell line and the Chinese hamster lung V79 cell line (Boos and Stopper 2000; Di Virgilio et al. 2004). Also, genistein was reported to be genotoxic by inducing DNA breaks as measured by the comet assay (Pool-Zobel et al. 2000). However, the Ames bacterial gene mutation test yielded no evidence of genistein mutagenicity (Bartholomew and Ryan 1980). The in vivo data on the ability of genistein to induce tumors in animal models are discrepant (Newbold et al. 2001; Thigpen et al. 2001). Furthermore, the therapeutic effect of genistein on breast cancer cells is dependent on the ratio of ERα/ERβ, which is high in MCF-7 cells. Hence, genistein treatment enhanced the viability of MCF-7 cells and the expression of antioxidant enzymes in them (Pons et al. 2016), which is in line with our results showing low cytotoxicity and no genotoxicity of genistein in these cells. This means that the consumption of genistein can be counterproductive in those patients who are diagnosed with breast cancer which harbors a high ratio of ERα/ERβ and who are receiving tamoxifen as an anticancer agent (Pons et al. 2016). Therefore, our findings along with previous reports suggest that the role of genistein as an enhancer or suppressor of tumor development is dependent on tumor type and possibly species.

Tartrazine belongs to a class of food dye that contains an azo group. In vitro and in vivo studies on the cytotoxic, genotoxic, and mutagenic effects of this compound are controversial and, in some cases, very unsatisfactory. Several studies have revealed that tartrazine does have genotoxic properties. This food dye induced chromosomal aberrations in Chinese hamster cells, *Muntiacus muntjac* fibroblasts, and bone marrow cells of rodents (Giri et al. 1990; Ishidate Jr et al. 1984; Patterson and Butler 1982). Furthermore, a significant toxic effect of tartrazine on the quality of human blood cells’ chromosomes was detected after an exposure of 72 h (Mpountoukas et al. 2010). Some studies also demonstrated a mutagenic effect of tartrazine in rodents. Tartrazine was found mutagenic in the rat studying its urine by the Ames test (Henschler and Wild 1985). However, rat feces proved negative by the same test (Münzner and Wever 1987). On the other hand, some other studies failed to find any evidence of mutagenicity or clastogenicity of tartrazine in vitro or in vivo (Elhkim et al. 2007). Similarly, tartrazine at a dose of 20, 200 ,or 1000 mg/kg did not elicit mutagenic or cytotoxic effects when administrated twice, with a 24-h interval, by oral gavage to mice. In this study, the in vivo gut micronucleus test and quantification of apoptic and mitotic cells were used to evaluate genotoxicity and cytotoxicity, respectively (Poul et al. 2009). Further in contrast to the positive genotoxic findings described above, we did not observe any statistically significant genotoxicity of tartrazine on MCF-7 cells using micronucleus test, although a tendency was evident (*p* for trend = 0.07).

Tartrazine cytotoxicity is an equally controversial issue. Soares et al. observed that tartrazine was not cytotoxic to human lymphocytes in the concentration range they tested (0.25–64 mM) (Soares et al. 2015). However, Mpountoukas et al. reported this compound to be cytotoxic to human lymphocytes at concentrations of 4 and 8 mM (Mpountoukas et al. 2010), which are within the concentration range used by Soares et al. Since both studies used MTT reduction-mediated assay to evaluate the cytotoxicity of tartrazine with overlapping concentration range on the same cell line, these discrepant results might be due to an inability of MTT assay to detect cells at an interphase between a metabolically active state and death (because MTT cytotoxicity assay is based on the reduction of MTT by mitochondrial enzymes), which could twist the cytotoxicity results. In contrast, LDH cytotoxicity assay is based on the release of intracellular enzymes to the culture medium after cell membrane damage (Fotakis and Timbrell 2006). Therefore, the LDH cytotoxicity assay that we used here is more robust to metabolic changes in the cell. Our finding of low cytotoxicity of tartrazine on MCF-7 cells has thus substantial significance in this field.

To put our cytotoxicity findings into a wider perspective, a useful example is bisphenol A (BPA). BPA is one of the high-production chemicals in the world that are used at least 3.6 million tons annually (Lu et al. 2013). BPA has a broad range of applications in the manufacture of plastic and epoxy resins that are used in food contact materials (Huang et al. 2012). BPA is also a synthetic xenoestrogen with notable cytotoxicity in LDH leakage assay; at 10^−4^ M, it has been reported to increase MCF-7-cell mortality more than 80% after 24-h incubation (Lei et al. 2017). In comparison, the maximum cytotoxicity of our test compounds in the LDH assay at the same concentration was less than 2.5%.

Phytoestrogens have been proposed as promising candidates for the prevention of estrogen-related cancers like breast cancer, prostate cancer, and, to some extent, endometrial and testicular cancer (Stopper et al. 2005). The possible adverse consequences caused by these compounds have seldom been considered, in contrast to synthetic xenoestrogens which have been seen mainly, if not exclusively, deleterious to health. Humans are highly exposed to dietary phytoestrogens because they are abundant in fruits, herbs, legumes, and vegetables (Peterson and Dwyer 1998). Most of the phytoestrogens belong to bioflavonoids, and the daily intake of bioflavonoids varies from 20 to 1000 mg (Stopper et al. 2005). Our present data support the view that the toxicological profile of a substance possessing estrogenic activity is not determined by its being a phytoestrogen or other natural xenoestrogens vs. a synthetic xenoestrogen. The synthetic xenoestrogen tartrazine fell between the two phytoestrogens 8-PN and genistein in its estrogenic activity but did not exhibit any greater cyto- or genotoxicity than either of them. This finding is in accordance with the data showing that a similar proportion of both natural and synthetic chemicals are carcinogenic in animals (Gold and Slone 1999).

## Conclusions

In the present study, 8-PN, genistein and tartrazine showed considerable estrogenic activity when assessed by a yeast reporter gene assay and MCF-7 cell proliferation assay. Strikingly, all these compounds exhibited low cytotoxicity and no genotoxicity in MCF-7 cells. Although the present study did not reveal any harmful effects, long-term exposure to these compounds has the potential to enhance the development of estrogen-dependent tumors. Thus, further research inclusive of long-term in vivo studies is warranted.

## Supplementary information

ESM 1 (DOCX 22 kb)

## Data Availability

The raw experimental data are available from AN upon request.
