# Supplementary material for: In vitro estrogenic, cytotoxic, and genotoxic profiles of the xenoestrogens 8-prenylnaringenine, genistein and tartrazine
Source: Environ Sci Pollut Res Int. 2021 Feb 1;28(22):27988–97. doi: 10.1007/s11356-021-12629-y (PMC8164609; doi:10.1007/s11356-021-12629-y)
Supplement: Supplementary file 1 — (DOCX 22 kb) [file 11356_2021_12629_MOESM1_ESM.docx]

**Supplementary tables S1 and S2**

**Table S1** Cytotoxicity screening test of test compounds in presence of 5% DMSO using *S. cerevisiae* BMA64/luc

| **Conc (M)** | **Correction Factor*** | | | | | |
| --- | --- | --- | --- | --- | --- | --- |
|  | **17 β-estradiol** | **8-PN** | **Genistein** | **Tartrazine** | **Progestrone** | **Tamoxifen** |
| 10^−14^ | 2.59±0.23 | 2.09±0.35 | 3.88±1.32 | 3.43±0.66 | 4.88±2.33 | 2.40±0.09 |
| 10^−12^ | 2.60±0.69 | 2.32±0.56 | 2.59±0.45 | 3.09±0.57 | 2.94±0.20 | 2.51±0.21 |
| 10^−10^ | 3.11±0.47 | 2.02±0.15 | 2.20±0.05 | 2.60±0.54 | 3.12±0.75 | 2.47±0.30 |
| 10^−8^ | 2.98±0.42 | 2.62±0.30 | 3.59±1.22 | 2.05±0.32 | 3.00±0.25 | 2.54±0.17 |
| 10^−6^ | 3.42±0.81 | 3.31±0.92 | 2.59±0.95 | 2.77±0.44 | 2.67±0.38 | 2.30±0.14 |
| 10^−4^ | 3.23±0.53 | 3.27±0.64 | 3.19±0.42 | 3.05±0.70 | 4.63±1.72 | 2.87±0.04 |

* The Correction Factor is the luminescence of the strain BMA64/luc with blank solvent divided by the luminescence of the control strain with the sample. Values higher than 2 are considered toxic (Leskinen et al. 2005). The values shown represent the mean ± SD of three replicates in a single experiment.

**Table S2** Cytotoxicity screening test of test compounds in presence of 5% ethanol using *S. cerevisiae* BMA64/luc

| **Conc (M)** | **Correction Factor*** | | | | | |
| --- | --- | --- | --- | --- | --- | --- |
|  | **17 β-estradiol** | **8-PN** | **Genistein** | **Tartrazine** | **Progestrone** | **Tamoxifen** |
| 10^−14^ | 1.26±0.22 | 0.98±0.12 | 1.58±0.23 | 1.14±0.23 | 1.22±0.23 | 1.39±0.31 |
| 10^−12^ | 1.13±0.18 | 0.92±0.14 | 1.30±0.22 | 1.10±0.26 | 1.19±0.40 | 1.23±0.16 |
| 10^−10^ | 1.13±0.04 | 1.03±0.16 | 1.07±0.04 | 1.36±0.30 | 1.34±0.10 | 1.28±0.25 |
| 10^−8^ | 1.11±0.06 | 0.97±0.04 | 1.18±0.24 | 0.95±0.09 | 1.32±0.25 | 1.50±0.27 |
| 10^−6^ | 1.08±0.05 | 0.94±0.14 | 1.27±0.42 | 0.94±0.12 | 1.35±0.21 | 1.21±0.32 |
| 10^−4^ | 1.12±0.12 | 1.05±0.21 | 1.59±0.17 | 0.99±0.08 | 1.22±0.48 | 1.35±0.25 |

* The Correction Factor is the luminescence of the strain BMA64/luc with blank solvent divided by the luminescence of the control strain with the sample. Values higher than 2 are considered toxic (Leskinen et al. 2005). The values shown represent the mean and range of two separate experiments. Each experiment was performed in triplicate.
